# An Updated Review of Soy-Derived Beverages: Nutrition, Processing, and Bioactivity

**DOI:** 10.3390/foods12142665

**Published:** 2023-07-11

**Authors:** Raquel Olías, Cristina Delgado-Andrade, María Padial, M. Carmen Marín-Manzano, Alfonso Clemente

**Affiliations:** Department of Nutrition and Sustainable Animal Production, Estación Experimental del Zaidín, Consejo Superior de Investigaciones Científicas, San Miguel 101, Armilla, E-18100 Granada, Spain

**Keywords:** bioactive compounds, industrial production, market survey, nutritional composition, soy beverages, soy milk

## Abstract

The global market for plant-based drinks is experiencing rapid growth driven by consumer demand for more sustainable diets, including vegetarian and vegan options. Soy beverages in particular are gaining popularity among individuals with lactose intolerance and milk protein allergies. They are considered an excellent source of high-quality protein, vitamin B, unsaturated fatty acids, and beneficial phytochemicals such as phytosterols, soy lecithins, and isoflavones. This review presents a comprehensive market survey of fifty-two soy beverages available in Spain and other European countries. The predominant category among those evaluated was calcium and vitamin-fortified drinks, accounting for 60% of the market. This reflects the need to address the nutritional gap compared to cow’s milk and meet essential dietary requirements. The review covers the technological aspects of industrial soy milk production, including both traditional methods and innovative processing techniques. Additionally, it analyzes multiple studies and meta-analyses, presenting compelling evidence for the positive effects of soy beverages on various aspects of health. The review specifically examines the contributions of different components found in soy beverages, such as isoflavones, proteins, fiber, and oligosaccharides. Moreover, it explores controversial aspects of soy consumption, including its potential implications for growth, puberty, fertility, feminization, and the thyroid gland.

## 1. Introduction

The plant-based drink (PBD) market is rapidly expanding across the world, substituting cow’s milk for many different reasons. This is related to the fact that consumers are seeking a more sustainable diet, substituting animal products, at least partially, with plant-based products. PBDs have been reported to exert beneficial effects on human health, being a major choice for lactose intolerance and for people allergic to cow milk protein. Demand for plant-based beverages has rapidly increased in recent years. A recent survey conducted as part of a European project (https://smartproteinproject.eu/) aimed to gain insight into consumer perceptions and preferences. The findings revealed that, following Germany, Spanish households emerged as the leading purchasers of plant-based drinks (PBDs) in Europe. Among the various PBD options, soy beverages ranked second in popularity, with oat-based drinks being the most widely consumed. From the nutritional point of view, soy-based drinks are an excellent source of high-quality protein, vitamin B, unsaturated fatty acids, as well as phytosterols, soy lecithins, and isoflavones. Although soy-based drinks are known as soy milk all over the world, the European regulation prohibits the use of the word milk for drinks that are not made from mammary secretions [1]. However, the Food and Drug Administration (FDA) has recently issued a recommendation concerning the labeling of plant-based dairy alternatives, describing the legality of using the term “milk” in such products [2]. Given the consumer’s increased familiarity with plant-based dairy alternatives, previous terminological restrictions are no longer necessary. Instead, clear labeling that highlights the nutritional properties of these products is recommended [3]. By accurately labeling and fortifying plant-based products already on the market, consumers will be able to evaluate the adequacy of vitamins and other micronutrients typically found in lower quantities compared to cow milk [4]. Accordingly, in this review, soy-based drinks are referred to as soy milk, soy drinks, or soy beverages.

This review aims to provide an updated analysis of various products available in the market by examining their nutritional composition. We have thoroughly examined the industrial production process, focusing on studying innovative methodologies employed to enhance the nutritional value of the end product. In order to provide a comprehensive understanding, we have extensively explored the fundamental nutrients present in soy milk, including protein, fats, carbohydrates, and isoflavones. Additionally, we have investigated how production processes influence the composition of these nutrients. Finally, this review offers a concise summary of the current understanding regarding the health benefits associated with consuming soy milk. Our analysis is primarily based on clinical and epidemiological research, allowing us to present an overview of the findings in a concise manner.

## 2. Market and Consumer Acceptance

A soy drink is a whitish liquid obtained by boiling ground soybeans, producing a colloidal suspension of plant material in water. The primary ingredients for this beverage include soybeans, water, emulsifiers, and occasionally additives. As the food industry continually innovates to enhance nutritional and sensory qualities to make it more attractive to consumers, the market is full of different beverages made from soybeans. However, a significant drawback is the absence of standardized information regarding the nutritional composition of these beverages. This review focuses on the analysis of commercial soy drinks, specifically 52 products from various brands (16), which are available not only in the Spanish market but also in several European countries. Based on the product label information, soy drinks were classified into five groups:

- Original soy drinks: those beverages declared only as “soy drink”;
- Fortified soy drinks: those drinks that contain added calcium (minimum 7.5% of nutrient reference values);
- Sugar-free soy drinks: those drinks that declare “no added sugars”;
- Light soy drinks: those drinks having 1.5 g of fat per 100 mL and 30% less energy value than the original product;
- Flavored soy drinks: those drinks with added flavors (chocolate, vanilla, caramel, cappuccino, nuts, cinnamon, and lemon).

These groups are not mutually exclusive, as certain commercial soy drinks possess more than one additional characteristic. For instance, some drinks may be both flavored and fortified, or sugar-free and light. Consequently, certain drinks have been included in multiple clusters to accurately reflect their diverse characteristics. Table 1 summarizes the nutritional composition of the commercial soy drinks studied, classified by category. A total of seven beverages met the criteria to be included in the original soy drinks category; four of them are supplemented with calcium (indicated in brackets). In the fortified soy drinks group, thirty-one beverages were included, among which four are labelled as “light”, four as “sugar free”, three as “high protein”, and seven as flavored. Twenty beverages were included in the sugar-free group, with six supplemented with calcium and one declared as “light”. In the low-fat group (named “light”), four beverages were included, all of them supplemented with calcium and only one beverage “sugar free”. Finally, thirteen beverages were included in the flavored soy drinks category, of which seven are supplemented with calcium, and two have a higher protein content.

Based on the labeling information, it can be observed that the average soybean content in all the beverages included in the survey is approximately 11% (*w*/*v*), except for the ones labeled as light drinks, which have a lower soybean content of 7.6%. This disparity in soybean content accounts for the reduced energy and protein levels found in low-fat soy drinks compared to the other beverages. The mean protein content of all products is 3%, with a range between 2.1% and 6%. Our laboratory conducted tests on eleven soy drinks included in the survey to determine their energy, protein (LECO), fat, and mineral content (Atomic Absorption Spectrometry) [5]. The results of the tests confirmed that the information provided on the labels was accurate and aligned with the actual composition of the drinks. In general, soy drinks have a closer protein content to cow’s milk (3.2%) than other PBDs [6]. The minimum protein content of soy drinks varies from one country to another, and although there are no established standards at the moment, the Soyfoods Association of America recommends a minimum of 3% of protein content for this product. Most soy drinks found in the Spanish market show a protein content closer to this reference value.

Among the samples collected for the market survey, approximately 26% of the commercial drinks claimed to be “sugar-free” (Table 1). However, it is important to note that the declaration of a value of 0 on the label does not necessarily indicate the complete absence of sugar but rather signifies a very low sugar content, approaching zero. Carbohydrates in soy drinks consist mainly of sugar and fiber. The sugar content declared on the label of these beverages is naturally present in the soybean and not added, except in the case of the flavored ones. These flavored soy drinks declared on the label the greatest values of carbohydrate content, around 6.3 g/100 mL, four times higher than the “light” category, probably due to the addition of sugar (not specified on the package) for sensorial reasons or due to the presence of sugars within the flavor additives. The inclusion of other carbohydrate sources in these products is probably the cause of the higher energy values found. The mean energy value of the non-flavored drinks, excluding the light ones, was 41 kcal/100 mL, while flavored drinks have an average energy value of 62 kcal/100 mL (Table 1).

To narrow the nutritional gap with cow’s milk and meet essential dietary needs, plant-based drinks (PBDs) are often supplemented or fortified with calcium and vitamins. In the case of soy drinks, it is common to add minerals like calcium and vitamins A, D, and B12 to enhance their nutritional content [7]. In this survey, it was found that 67% of the fifty-two analyzed beverages were supplemented with calcium. Vitamin A was present in 46% of the drinks, vitamin D in 71%, vitamin B12 in 26%, riboflavin in 23%, and only one beverage contained vitamins E, C, thiamine, niacin, B6, and folic acid. One of the primary concerns when replacing cow’s milk with a plant-based drink (PBD) is the calcium supply. Traditional soy milk has a relatively low calcium content of 25 mg/100mL compared to cow’s milk, which contains 120 mg/100 mL [8]. In our survey, we observed that all the drinks labeled as fortified indeed contained added calcium, and only a limited number of them included other essential minerals like phosphorus or magnesium, as well as vitamins A and D. It is noteworthy that 48% of the drinks not specifically labeled as fortified still had added calcium. This indicates that a significant portion of soy drinks in the market, despite not being marketed as fortified, still incorporate additional calcium to enhance their nutritional profile.

**Figure 1:**
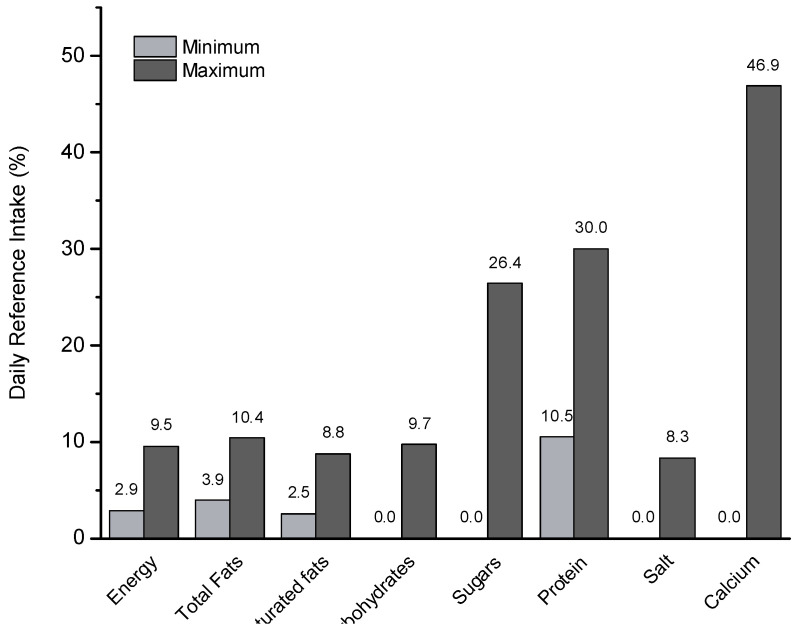
Contribution (%) of a 250 mL portion of soy beverage to the daily reference intake of nutrients in healthy adults according to EU regulation 1169/2011.

Figure 1 illustrates the contribution (*n* = 52) of a 250 mL portion of soy drink to the daily reference intake of different nutrients established by the European Union (EU regulation 1169/2011). A 250 mL glass of soy drink could contribute between 3 and 10% of the energy, fat, and carbohydrates needed daily in healthy adults. A 250 mL portion provides up to 30% of daily protein needs. When properly fortified, almost 47% of daily calcium needs are covered. However, special attention must be paid to the sugar supply, since a portion of the flavored product could contribute more than a quarter of the daily recommended intake of sugars.

The market survey presented in this review reveals a wide range of options available in the soy drink market, ranging from basic presentations with no additives to comprehensive nutritional products fortified with minerals and vitamins. The category of “fortified soy drinks” was found to be the most prevalent, indicating that consumers are attracted to beverages with an enhanced nutritional profile. However, it is important to note that the labeling of soy drinks is sometimes incomplete when it comes to declaring the nutritional composition. This includes aspects such as fortification, the quantity of the main ingredients, the presence of added sugar, and other factors that can significantly impact the final composition of the product. This lack of standardized information may pose challenges for consumers seeking to make informed choices about the soy drinks they purchase.

## 3. Industrial Production

### 3.1. Traditional Methods

**Figure 2:**
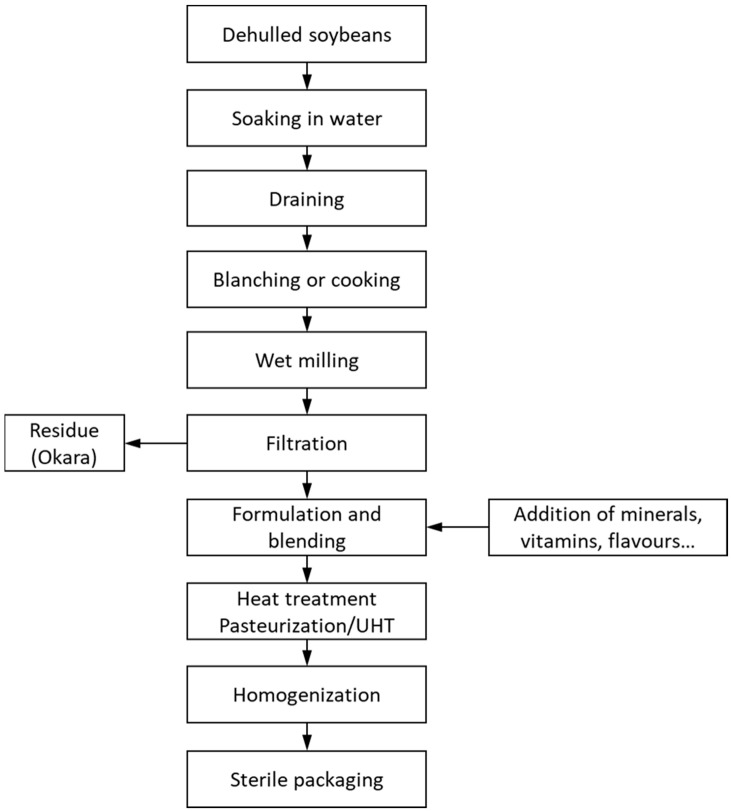
General diagram for soy beverage production.

The nutritional characteristics of soy milk can be influenced by various factors, including the quality of soybean material, the processing methods employed, and the storage conditions. Traditionally, soy milk is prepared using a straightforward process that involves soaking soybeans, wet grinding them, boiling the mixture, and then filtering it to obtain the soy drink (Figure 2; adapted from Aydar et al. [9]). This traditional method has two main drawbacks; the resulting soy drink has a strong beany flavour and a short shelf life [10]. Several procedures have been developed to enhance the extraction yield of the protein, to improve the content of bioactive compounds, and to inactivate LOX, responsible of the beany flavor, and anti-nutritional factors including trypsin inhibitors (TIs) that are primarily responsible for the low digestibility of legume proteins [7,11,12]. Other methods, such as homogenization or heat treatment, have been implemented to improve stability and to extend the product’s shelf life [13]. Although some differences between processes can be found, a standard diagram of the current industrial-scale soy drink manufacturing process is shown in Figure 2. Soybeans require some pre-treatments, such as dehulling, soaking, and blanching, to improve the nutritional and organoleptic characteristics of the resulting soy drink. Soybeans can be soaked in water or in an alkaline solution. If a sodium carbonate solution is used (0.4 M Na_2_CO_3_), a significant decrease in the beany flavor is achieved, compared to water soaking. Additionally, better viscosity, yield of protein and fat, and easier processing are related to alkaline soaking [14]. To eliminate the beany flavor caused by the action of LOX over fatty acids, the soybeans can be blanched. Blanching is a cooking technique that involves briefly boiling or scalding food items in hot water and then transferring them to cold water to stop the cooking process. Blanching (0.5–10 min at 70–85 °C) effectively reduces the production of off flavor. Additionally, this process contributes to a significant decrease in trypsin-inhibitory activity (TIA) by approximately 25–50% [15]. Reducing TIA improves the digestibility of soybean proteins since protease inhibitors (KTI and BBI) present in the seeds form stable complexes with the digestive enzyme trypsin. Hot water blanching, carried out at temperatures between 80 and 100 °C for a duration of 2–10 min, followed by hot grinding, plays a role in reducing the beany flavor of soybeans. However, it is crucial to carefully manage the process to maintain a desirable balance between diminishing the beany flavor and preserving the non-beany flavors [16]. Once the soybeans have been blanched and ground, the resulting slurry must be filtered to separate residues from the beverage. Product formulation is the next step, in which other ingredients and additives, such as minerals, vitamins, flavorings, salt, oil, food colorings, or stabilizers are added to the liquid [13].

To extend the shelf life, the soy drink can be pasteurized or ultrahigh-temperature-treated (UHT). Heat not only destroys microorganisms but inactivates, at least partially, protease inhibitors and LOX, which negatively affect the nutritional and organoleptic properties of the final product [17,18]. Finally, homogenization increases the physical stability of the final product by decreasing the particle size. It also improves the viscosity and appearance, making the texture of the beverage more similar to that of cow’s milk [13,19].

### 3.2. Innovative Methods

In the last few years, several techniques related to the soy milk manufacturing process have been studied, mainly focused on inactivating enzymatic activities or increasing extraction yields, product stability, and shelf life (Table 2). Sustainability is also an important factor that has been considered when novel technologies are developed. One of the goals of the food industry is to reduce energy consumption and maximize raw material value. As an example, ultrasound-assisted technology, ultra-high-pressure homogenization, and enzyme-assisted extraction, among others, are considered as eco-friendly technologies [20].

During the production of soy milk, certain vital nutritional components such as proteins and isoflavones often end up being lost in the solid residue known as okara, resulting in incomplete incorporation into the soy milk. To address this, several innovative technological methods are being developed to enhance the extractability of these nutritional components. The objective is to improve the retention and utilization of these valuable constituents in soy milk production. Ultrasound treatment, for instance, has been found to increase product stability and improve extraction yields of proteins, oils, and solids. Additionally, it helps in reducing off flavors by inactivating lipoxygenase (LOX). Ultrasound treatment causes the soy slurry particles to disintegrate, shifting to a smaller particle size (in the range 0.3–1.1 μm). The increased protein extraction is mainly due to an improved solubility and not cell disruption [23,24]. When applied as a pre-treatment to soybeans, ultrasound leads to a substantial rise in the isoflavone content found in the resulting soy drink. This increase is attributed to the release of isoflavones from either the soybean cell walls or the protein–polyphenol complex [22]. Moreover, when ultrasounds are combined with microwave treatment, the nutritional value of the final product is further enhanced. This combination results in reduced levels of trypsin inhibitor (TI) and lipoxygenase (LOX) activities, along with modifications in the protein structure. These collective effects contribute to improved protein digestibility, ultimately enhancing the overall nutritional quality of the product [25,26]. Utilizing low-intensity pulsed electric fields as an extraction-aiding technology during the manufacturing process facilitates the modification of both the total content and profile of isoflavones. This desirable outcome is attributed to the increased activity of β-glucosidase enzymes, which promotes the hydrolysis of malonyl and acetyl structures. Consequently, this enzymatic process leads to elevated levels of aglycones and a higher overall content of isoflavones [27]. Applying an enzymatic pre-treatment to soybeans leads to a reduction in okara residues and an increase in protein and isoflavone content in the resulting soy drink. This pre-treatment involves enzymatic hydrolysis of the polysaccharide cell wall using a mixture of carbohydrases, including cellulase, hemicellulase, arabinanase, β-glucanase, and xylanase. As a notable effect, this enzymatic process significantly boosts the protein recovery rate in the final soy milk [28]. 

Numerous studies are currently underway to develop effective strategies for eliminating lipoxygenase (LOX) activity and reducing anti-nutritional factors such as trypsin inhibitors (TIs) and phytates. Microwave treatment, in addition to improving extractability, helps decrease LOX and TI activities by elevating the temperature [17]. Manothermosonication, which combines ultrasonic waves with mild heat and pressure, has proven to be an effective technique for inactivating enzymes like LOX, peroxidase (POD), and TI [21]. Similarly, a radio-frequency pre-treatment has been found to enhance the sensory properties of the beverage while reducing levels of anti-nutritional factors such as LOX, urease, and TI [29]. High-temperature pressure cooking has also been explored and has shown improvements in digestibility and sensory attributes compared to beverages treated with conventional cooking methods [31]. Non-thermal processing techniques are also being studied to minimize thermal degradation of nutrients while improving the nutritional quality. The addition of natural compounds such as tea polyphenols (TPs) and stevioside has been reported to inactivate TI [32,33,34]. These compounds interact with amino acids near TI’s reactive sites, reducing its protease-inhibitory activity. Epigallocatechin gallate, a main tea polyphenol, interacts with TI, inducing conformational changes that reduce the inhibitory protease activity [33]. Such an interaction is very complex; TPs can accelerate the aggregation process of soy proteins after heating [36], and the decrease in TI activity could be caused not only by the structural changes in KTI but also by the aggregates induced by TPs. Hydrogen bonds and hydrophobic interactions between TP and BBI residues may be the main reason for the decrease in chymotrypsin-inhibitory activity [32].

To address the naturally low calcium content in soy drinks, calcium fortification is commonly employed. However, novel methods are being developed to enhance calcium extraction from soybeans, alongside exploring alternative fortification techniques. One such method, tested at the laboratory scale, involves the use of the anaerobic grinding method combined with calcium lactate as the calcium source. This approach has shown promising results in producing calcium-enriched soy milk with high acceptability, while keeping sensory properties intact. These advancements aim to provide a calcium-rich soy beverage without compromising its sensory appeal [8]. Anaerobic grinding aims to minimize the oxidation of lipids and other components in soybeans, which can lead to off flavors, color changes, and nutrient losses. To improve mineral absorption, it is crucial to address the presence of phytic acid. This antinutrient has the tendency to form complexes with minerals like calcium, which are not easily solubilized and ionized, even under the low-pH conditions encountered during gastric transit. Consequently, the formation of these complexes limits the absorption of minerals, hindering their bioavailability and utilization by the body. Taking steps to reduce phytic acid levels can significantly enhance the absorption of essential minerals and promote overall nutritional benefits [37]. The addition of free phytase, an enzyme that catalyzes the hydrolysis of phytates, has been shown to effectively enhance mineral absorption. However, this approach is not economically feasible for the industry. To address this, researchers are exploring phytase immobilization techniques using various matrices at the laboratory scale. These methods show promising results and offer potential solutions to improve mineral bioavailability without incurring high costs for the industry [35].

In conclusion, the soy milk market is experiencing rapid growth, driven by consumer demands for highly nutritious products and a sustainable food industry. To meet these demands, the food industry is actively exploring new strategies for soy beverage production. This includes the investigation of effective and advanced processing technologies to address challenges such as shelf life extension, emulsion stability, nutritional completeness, and sensory acceptability. In the coming years, we can expect the development of tailor-made soy milk products that are not only palatable but also nutritionally suitable for various population sectors. These advancements aim to provide consumers with a wide range of appealing and nutritionally adequate soy milk options.

## 4. Nutritional Properties of Soy-Derived Beverages

### 4.1. Protein Composition

Soy milk consists of a protein dispersion and a protein/lipid emulsion. The majority of proteins found in soybean seeds are extracted during the preparation of soy milk. While the protein content is primarily influenced by the bean variety and environmental factors during cultivation, the manufacturing process also plays a significant role in determining the final composition of the soy drink [38]. Soybean proteins represent 30–60% on a dry weight basis and soy milk contains around 3–4% protein. The protein content of the soy beverages evaluated for this review is in agreement with this percentage, excluding the case of the “light” category, where a lower average content of 2.3% was reported for the group (Table 1). Most of the proteins present in the seeds are storage proteins. The major storage proteins of soybeans, glycinin (11S) and β-conglycinin (7S), represent 80% of soybean protein extracted in soy milk. Glycinin is an hexameric protein, and its subunit (A-SS-B) is composed of one acidic polypeptide (A) and one basic polypetide (B) linked by a disulphide bond (SS). β-conglycinin is a trimeric glycoprotein including α’, α, and β subunits. Half of α’, α subunits are SS-linked together or with protein P34. P34 exists either as a monomer or as α’/α−P34. Apart from these storage proteins, soy milk contains several albumins, including LOX, β-amylase, lectin, KTI, and BBI, with different biological functions.

**Figure 3:**
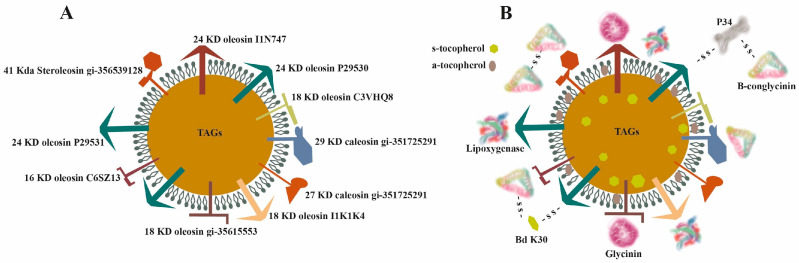
(**A**) Schematics of a purified soybean oil body model, including a core of triglycerides (TAGs) in yellow, a layer of phospholipids in grey, and oleosins integrated. (**B**) Interaction of external proteins with intrinsic proteins of oil bodies (adapted from [39]).

In soybean cells, the storage proteins β-conglycinin and glycinin and other minor proteins such as P34 are deposited in protein vacuoles (PVs). Other proteins are associated with oil bodies (OBs). OBs have a matrix core of triacylglycerols (TAGs) covered by a layer of phospholipids with seven embedded oleosins that play a major role in the size of the OB and can interact with other proteins on the surface, two caleosins and one steroleosin [39] (Figure 3). Because of the surface charge of oleosins, OBs do not coalesce or aggregate; their charged surfaces repel each other and prevent aggregation. 

The interaction between proteins and OBs is essential to determine the protein distribution in the liquid and nutritional quality of the soy milk. After standard aqueous extraction, PVs are disrupted, while OBs, which are quite stable even at high temperature, acquire a second protein layer consisting mainly of LOX, glycinin, β-conglycinin, and Bd 30K/P34 [40]. Soy milk protein subunits are unevenly distributed among soluble and particulate proteins. Soluble proteins are hydrophilic. The α and α′ subunits of 7S, the acidic (A) subunit of 11S, and the BBI of whey soybean proteins (WSPs) are commonly found in the soluble protein fraction. By contrast, other WSPs, such as the β subunit of 7S and the basic subunit (B) of 11S, are abundant in particulate proteins.

Cooking raw soy milk results in denaturalization and polymerization of proteins to form other particles that vary in size, composition, and morphology. These particles will influence the stability of the soy milk product. In the cooking process at high temperature, the outer proteins are free from the inner core of OBs, resulting in their liberation to the floating fraction of the soy milk [41].

To avoid the enzymatic oxidation of unsaturated fatty acids by LOX, responsible for the beany flavor of traditional soy milk, soybean is milled at high temperatures to inactivate the enzymatic activity. Milling at high temperatures (>70 °C) leads to an increase in the amounts of precipitate [42]. This has been mainly attributed to the denaturation of β-conglycinin at temperatures above 70 °C [43]. Although protein composition does not change at different milling temperatures, the protein content gradually decreases at temperatures above 80 °C, indicating a partial insolubilization or incomplete solubilization of proteins [42]. Many Western manufacturers apply a blanching process to inactivate LOX activity. This includes soaking the beans in hot water (>80 °C) for more than five minutes before hot grinding and extraction, the removal of the okara (solids), and the cooking process. This process results in not only the inactivation of LOX but also in changes in the size of particles that tend to precipitate, making the final product less stable with a shorter shelf life. Blanching alters the denaturation and aggregation mechanism of soy milk particles, trapping parts of oil bodies into particulate proteins (polymeric aggregates formed from protein subunits). However, the characterization of protein particle aggregation in blanched soy milk is still unclear [41].

The use of high-pressure treatment instead of heating revealed that soy proteins were dissociated into subunits, some of which associated into an aggregate and became insoluble, increasing the viscosity of the soy milk [43]. Many studies have tried to elucidate the composition of soy milk particles. All the modifications experienced by the proteins and OBs during the manufacturing will affect the posterior digestibility of the proteins. Even heating soy milk before consumption alters the OBs and modifies its protein composition and structure, allowing a better proteolysis by digestive enzymes and the release of more bioactive peptides than in non-heated soy milk [44].

The protein composition of soy milk after the manufacturing process is not well-defined. A study on 15 different commercial soy milks using a label-free quantitative proteomics approach revealed variations in the protein composition influenced by the processing as well as the genetic background of the soy cultivars [45]. The protein profile of soy milk reflected soybean protein composition, which is rich in proteins with storage functions. Apart from the storage proteins, the soybean contains around 20–30% bioactive proteins that include beta-amylase, cytochrome c, lectin, LOX, urease, KTI, and BBI. Up to 23 different proteins were identified in commercial soy milks, including multiple isoforms of glycinins, beta-conglycinins, oleosins, sucrose binding proteins, and late embryogenesis-abundant proteins, with a beta-conglycinin (P0DO16) having the highest average mass (%). The highest variability in terms of protein amount between commercial soy milks was found for beta-conglycinins, while glycinins and oleosins have less variability between different samples. From proteomic data collected, the amino acid content of soy milks was indirectly estimated. Differences between samples were observed in the amino acid composition; all analyzed commercial soy milks showed a relatively low content of histidine, methionine, tryptophan, and cysteine [45].

Protein quality is primarily determined by factors such as amino acid composition, protein digestibility, and amino acid bioavailability. The Food and Agricultural Organization of the United Nations/World Health Organization (FAO/WHO) recommend the use of the Digestible Indispensable Amino Acid Score (DIAAS) to assess protein quality. DIAAS takes into account the true ileal digestibility of each indispensable amino acid, relating the amount of ingested protein to the amount of amino acid absorbed by the body in the small intestine. A recent review addressing the protein quality of various soy products highlighted the limited scientific evidence available specifically on soy milk, despite its widespread consumption [46]. Additional research is necessary to explore the complex process of human digestion and assess the bioavailability of peptides and amino acids from commercial soy milk produced through different manufacturing processes. The authors of the review noted that most soy products generally exhibit high protein quality scores. However, they also noted that most studies evaluating the Digestible Indispensable Amino Acid Score (DIAAS) have utilized different animal models or various in vitro methods, which presents a challenge when comparing results. In this sense, the recently developed in vitro digestion harmonized protocol, INFOGEST [47], will be very useful to evaluate the protein quality of the different soy drinks available in the market. A recently published analytical workflow will allow the assessment of protein digestibility and DIAAS calculation of the different soy drinks available in the market [48].

### 4.2. Fatty Acid Composition

Soybeans are abundant in lipids, making up approximately 18–24% of their total dry weight, while soy milk contains around 2% lipid content. Consequently, soy milk is considered a low-fat beverage due to its relatively low lipid content compared to the original soybeans. The soy drinks listed in Table 1 generally align with this lipid content, except for the “light” category, which showed a lower value of 1.2%. Indeed, the decrease in lipid content observed in the "light" category of soy drinks is likely attributed to the utilization of a reduced amount of soybeans during the manufacturing process to obtain the final product.

The majority of fatty acids (FAs) found in mature soybeans are unsaturated fatty acids, accounting for approximately 85% of the total. Among them, linoleic acid (C18:2n C6) is the most abundant, making up 50% of the total fatty acid composition. The fatty acid profile of traditionally obtained soy drinks closely resembles that of soybeans, with significant amounts of linolenic acid (C18:3 n3), oleic acid, palmitic acid, and stearic acid [49]. All these unsaturated fatty acids are susceptible of oxidation during soy milk preparation since this normally implies the use of high temperatures. However, using the traditional method of soy milk preparation, only slight differences in the FA profile were observed due to small increases in palmitic and stearic acids and the appearance of cis-vaccenic acid (C18:1 n−7), a positional isomer of oleic acid (C18:1 n−9) not present in soybeans. Moreover, this procedure does not promote trans-FA formation [49].

As mentioned earlier, the storage of oil in soybeans occurs within discrete spherical organelles known as oil OBs, lipid bodies, oleosomes, or spherosomes. These organelles enclose triacylglycerols (TAGs) within a phospholipid monolayer, which is further covered by a protein layer, providing exceptional stability to the structure (as depicted in Figure 3). These spherical organelles function as a natural emulsion of lipids, preventing coalescence or aggregation.

However, there is limited information available regarding the fate of fatty acids during the manufacturing process of soy milk, as most of the research has primarily focused on the purification of OBs and understanding the release of proteins from these spherical organelles [50,51]. Further investigation is required to explore the destiny of fatty acids during soy milk processing, including their potential interactions with other components and their impact on the overall stability and nutritional composition of the final product. As mentioned above, blanching causes changes in the soy milk particle size because the components that are normally released are trapped within the particulate, increasing the mass of this portion to the extent that it is 10% higher than that of traditional soy milk. The particulate and soluble fractions of traditional soy milk consist of a negligible amount of lipids (~4–5% of the total fat). By contrast, the ratio of lipid content in the floating fraction of traditional soy milk accounts for over 95% of the total fat [41].

Soy milk is also a good source of phospholipids, the major components of the cell membrane, with large contents of phosphatidylserine, phosphatidylglycerol, phosphatidyl ethanolamine, and phosphatidylcholine compared to cow milk [52]. Phospholipids are involved in many physiological functions such as anti-inflammatory activity, reducing cholesterol absorption and the risk of cardiovascular diseases.

Gaining new knowledge regarding how heat affects the structure of oleosomes during the preparation of soy milk is crucial. This understanding is significant as it directly impacts the release of TAGs (triacylglycerols) during the process of digestion. By investigating the structural changes induced by heat, we can better comprehend the implications for nutrient availability and digestion of soy milk [44].

### 4.3. Carbohydrates

Despite the high amount of carbohydrates contained in soybeans (≈30%, including starch and non-starch polysaccharides), the analysis of the carbohydrate content of commercial soy beverages has revealed a low total carbohydrate content ranging from 0.26% to 5% (g/100 mL) [53], an interval similar to that found in our market survey, except in the case of flavored beverages (Table 1). Soy milk has no lactose and low amounts of starch, glucose, and fructose, sucrose being the main carbohydrate (97%). The low amount of total carbohydrates may be due to the concentration of soybean used in the production of the beverage, usually around 7–9% of whole or dehulled seeds [53].

The carbohydrate content in a soy drink is influenced by its processing method. To determine the most effective approach for obtaining the carbohydrate fraction in the final product, Li et al. [54] conducted a comparative study using the traditional processing method with three variants: (i) direct grinding in water without soaking; (ii) including a previous soaking step; (iii) including soaking, freezing, and air-drying of seeds. The study concluded that the soy beverage from soaked soybean has the highest content of total carbohydrates (21.6 mg/mL) and non-digestible oligosaccharides such as raffinose and stachyose, compared to the soy beverage from untreated soybean, which contains the lowest amount of total carbohydrates (16.6 mg/mL) and non-digestible oligosaccharides. On the other hand, the soaking–freezing–air-drying procedure showed a lower content of oligosaccharide extraction compared to soaked soybeans, but higher than untreated soybeans. In addition, the oligosaccharide content of the soy beverage from the soaking–freezing–air-drying procedure showed a gradual trend of enhancement when increasing the number of freezing days to 4 days. These findings, together with a similar protein content and a higher Fe content than the soaked soy beverage, suggest that this method would be a good alternative for the production of soy beverage in terms of oligosaccharide occurrence. Soybean oligosaccharides have been reported to have prebiotic effects and are linked to several health benefits, such as lowering blood cholesterol levels, improving mineral absorption, and preventing some types of cancer [55] (see Section 5.7 for further details).

### 4.4. Isoflavones

Soybeans contain a significant amount of isoflavones, which are linked to health benefits. In 1999, the US Food and Drug Administration approved a health claim for the cholesterol-lowering effects of soybeans, attributed to their isoflavones. The isoflavone content in soy milk is influenced by many factors such as soybean variety, method of processing, processing temperatures, duration of heating, and addition of other components [27,56,57]. The main isoflavone compounds, genistein, daidzein, and glycitein, occur in four chemically different forms as aglycone, β-glucoside, acetyl-β-glucoside, and malonyl-β-glucoside. Isoflavones contained in soybeans and soy milk are mainly 6’-O-malonyl-β-glucosides, which are partly transformed to 7-O-β-glucosides and 6’-O-acetyl-β-glucosides upon thermal processing. The bioavailability of isoflavones can be affected by their chemical forms in foods and their stabilities during processing. Many different studies have reported that heat processing causes de-esterification of malonyl- and acetyl-glucosides to their underivatized β-glucosides, explaining the presence of mostly glycosides in soy milk. Heating substantially decreased daidzein and glycitein contents in soy milk [58]. The extent of loss of the isoflavone aglycones in soy milk as affected by thermal processing was found to decrease in the order daidzein > glycitein > genistein. On the other hand, heat treatment may cause variation in the genistein content of soy milk depending on the temperature and time applied. Apparent increases may occur under optimum heating conditions, possibly owing to the conversion of genistein to genistein. Xu and Chang (2009) [59] demonstrated how UHT processing of soy milk, which implies heating quickly to a high temperature (143 °C) in a short period of time (≤1 min), had different effects on isoflavone composition of soy milk depending on the variety of soybean used. On the other hand, severe heating causes chemical reactions such as Maillard-type reactions and autodegradation, resulting in a decrease in the genistein content [58]. Recent studies confirmed these findings, comparing three different processing methods with 15 different soybean varieties [57].

## 5. Bioactive Properties of Soy Milk

**Figure 4:**
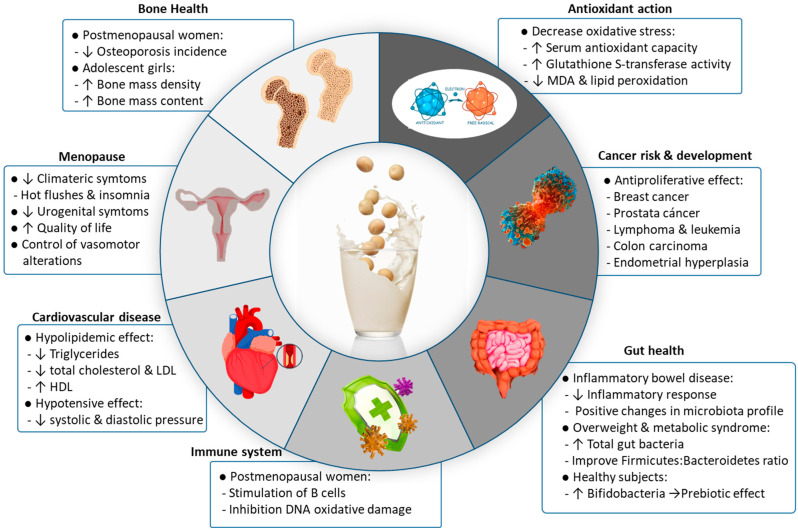
Biological actions attributed to soy beverages supported by scientific evidence.

Since soy milk is a good source of protein, micronutrients, and phytochemicals, different health claims have been proposed [19,60,61] (Table 3; Figure 4). The main health-beneficial properties associated with soy milk consumption are described below.

### 5.1. Antioxidant Activity

Despite the impact of processing on bioactive compounds with antioxidant activity and the predominance of hydrophilic molecules retained after the aqueous extraction process, soy milk retains a significant level of antioxidants. This antioxidant-rich composition has the potential to provide beneficial effects in various chronic diseases, including atherosclerosis, cardiovascular disease, cancer, and diabetes. [69]. Phytoestrogens, including genistein, daidzein, and glycitein, are recognized as key compounds responsible for various effects. These molecules, structurally analogous to natural estrogens, exhibit scavenging capabilities against free radicals generated during the oxidation of proteins, lipids, nucleic acids, and DNA [9], which is the main mechanism of action behind many of the biological activities attributed to the soybean. The lowering of oxidative stress has been suggested as one of the more efficient pathways to reduce the risk and progression of some disease conditions. According to the study by Onuegbu et al. [62], the daily consumption of 500 mL of soy milk by a group of healthy individuals for 28 days resulted in significant increases in serum total antioxidant capacity (measured using the FRAP method) and glutathione S-transferase (GST) activity. Concurrently, there was a reduction in malondialdehyde (MDA), which suggests a decrease in lipid peroxidation. Concerning these usual antioxidant methods, GST catalyzes the conjugation of l-glutathione to 1-chloro-3-4 dinitrobenzene through the thiol group of the glutathione. The formation of the glutathione conjugate is proportional to the enzyme activity. In the case of the lipid peroxidation product MDA, a spectrophotometric method based on the pink-colored product formed when MDA is heated with 2-thiobarbituric acid under an alkaline condition can be applied. A recent investigation has stated that total polyphenol content (measured by the traditional Folin–Ciocalteau method) detected in soy milk after gastrointestinal digestion is similar to that found in cow’s milk, whereas antioxidant activity measured by ORAC, ABTS, and FRAP methods was significantly higher [70]. It is widely recognized that the determination of antioxidant activity depends on the reaction mechanism employed; therefore, combining multiple tests can offer a more reliable evaluation of antioxidant profiles. The ABTS and the ORAC procedures operate on similar principles, both assessing free radical scavenging ability, with the particularity that the latter assesses the ability of a compound to neutralize free radicals existing in physiological media, whereas the former is based on the generation of radicals not naturally occurring in the organism. The FRAP method works with a different mechanism of reaction by measuring the ferric reduction capacity of the sample [71]

### 5.2. Cardiovascular Disease Risk Factors

In recent years, there has been increasing interest in the potential hypolipidemic effect of soy milk, primarily due to certain components present in it. These components, including specific proteins, polyunsaturated fatty acids, saponins and phytosterols, lecithins, and isoflavones, have been investigated for their potential impact on lipid metabolism [72]. It has been established that a soy protein intake of at least 25 g/day, which could be provided by around 400 mL of a high-protein soy milk, can reduce blood cholesterol due to an upregulation of LDL receptors and to an increase in bile acid fecal excretion. Although FDA has been considering retracting the heart health claim for soy protein, a recent meta-analysis concluded that it significantly reduced LDL cholesterol by approximately 3–4% in adults [73]. As a synergic mechanism, isoflavones such as genistein and daidzein seem to be capable of stimulating mRNA expression of genes and/or activating enzymes participating in fatty acid β-oxidation and decreasing the expression of genes and/or activity of enzymes involved in lipogenesis [74]. Several in vivo models have shown the favorable effects of soy milk consumption on reducing triglycerides, total cholesterol, and/or LDL cholesterol, as well as increasing HDL cholesterol [75,76]. However, evidence derived from randomized clinical trials to demonstrate the hypocholesterolemic effect of soy milk is still inconsistent [77], and more studies are necessary.

Clinical studies have confirmed that soy protein consumption has a hypotensive effect, with a significant reduction in diastolic blood pressure [78]. Soy milk has also been documented to have a similar action in a more attenuated way, which is not negligible, since any reduction in the systolic blood pressure by just 2–5 mmHg may reduce stroke and coronary heart disease by 6–14% and 5–9%, respectively [69]. Rivas and colleagues [63] compared the effects of the 3-month consumption of one liter per day of soy milk vs. cow’s milk in men and women with mild to moderate hypertension. This study demonstrated a significant hypotensive action of soy milk which correlated with the urinary excretion of genistein. A meta-analyses study showed that systolic and diastolic blood pressure is reduced by approximately 2.5 and 1.5 mmHg, respectively [69]. The existence of peptides with inhibitory activity of angiotensin converting enzyme (ACE) after simulated gastrointestinal digestion of soy milk [79] and soy milk proteins [80] has been reported. These peptides may help to reduce blood pressure by inhibiting the production of angiotensin II, a hormone that constricts blood vessels and raises blood pressure. ACE inhibitory peptides derived from soy milk obtained by enzymatic hydrolysis with commercially available bacterial proteases have been proposed as bioactive components in food supplements for patients with hypertension [81].

Endothelial dysfunction is a type of coronary artery disease that narrows arteries. The action of isoflavones in this disorder has been contradictory and no differences in arterial stiffness were detected in a randomized, placebo-controlled crossover trial developed in hypercholesterolemic men and women after consumption of soy milk compared with dairy milk for five weeks [82]. Some meta-analyses showed that soybean isoflavones improved endothelial function and reduced arterial stiffness in postmenopausal women without affecting inflammatory biomarkers [83,84]. However, evidence derived from randomized clinical trials to demonstrate the hypocholesterolemic effect of soy milk is still inconsistent [77].

### 5.3. Cancer Risk and Development

The proteins and isoflavones found in soy milk have been associated with a potential protective effect against certain types of cancer, particularly hormone-dependent cancers. This effect is attributed to the presence of phytoestrogens, such as genistein and daidzein, as well as their active metabolites [9]. Genistein has shown promising chemotherapeutic properties against various types of cancers. One interesting aspect of genistein is its potential for synergistic effects when combined with common anticancer drugs such as adriamycin, docetaxel, and tamoxifen [85]. Daidzein is a strong antioxidant with the ability to protect against breast and prostate cancer. Moreover, unlike genistein, daidzein follows a metabolic pathway producing compounds with estrogenic action, equol being one of the more active metabolites. Hod et al. [86] described the anticancer properties of equol mediated via reduced expression of matrix metalloproteinases, which results in impaired metastasis characteristics in breast cancer cells and enhanced demethylation of BRCA1 and BRCA2 promoters, which reduce the risk of the early onset of breast cancer. Other related studies suggest that equol’s anticancer properties are mediated by induced apoptosis and their anti-proliferative effects on radioresistant breast cancer cells.

Although in a lower amount than genistein and daidzein, soy milk contains another bioactive isoflavone, glycitein. Despite having a lower binding ability to estrogen receptors, glycitein has the ability to trigger a more pronounced estrogenic response. This is attributed to its enhanced bioavailability compared to genistein. Furthermore, similar to daidzein, the metabolism of glycitein results in the formation of compounds with even higher estrogenic activity [87].

It is widely recognized that breast cancer incidence rates in soy food-consuming countries are much lower than in Western ones. A high soy consumption is associated with approximate a one-third reduction in breast cancer risk but, interestingly, considerable data suggest that for soy to perform this action, the intake must occur during childhood and/or adolescence. Case–control studies evidence that increased soy intake early in life is related to 25–60% reductions in cancer risk [69,88]. This “early intake” hypothesis is supported by the fact that clinical studies in adults have shown no effects of soy protein or isoflavone supplements on markers of breast cancer risk [89,90]. The Shanghai Women’s Health Study provided interesting findings on the importance of timing in the protective effect of soy intake on breast cancer risk. More than 70,000 women were recruited and monitored for a period of around 13 years, after which 1034 breast cancer cases were detected. The women were categorized into three groups based on their soy protein intake: low, medium, and high consumption. The data indicated that a high intake of soy protein during both adolescence and adulthood was significantly associated with a reduced risk of breast cancer. However, consuming higher amounts of soy exclusively during adolescence and not during adulthood resulted in a moderate protective effect that was not statistically significant [91]. Conclusions drawn from a European prospective investigation into cancer and nutrition named EPIC, which included a good portion of vegetarians with high soy consumption, also support the “early intake” hypothesis. This research found no association between isoflavone intake and breast cancer risk, likely because the heavy soy consumers in this cohort began eating soy later in life, when they adopted a vegetarian diet [92].

Fraser et al. [64] conducted a study to investigate the potential links between soy milk, other soy products, dairy milk, other dairy foods, and the risk of breast cancer in a cohort of 52,795 North American women who had no prior history of cancer. Their findings revealed a noteworthy positive association between dairy consumption, particularly milk, and the risk of breast cancer. However, this risk decreased when median intakes of dairy milk were replaced with soy milk, suggesting a potential protective effect of soy milk in this context. Tan et al. [65] conducted an unmatched hospital-based case–control study in a Malaysian population considering a cohort of 3683 cases (breast carcinoma) and 3980 controls (healthy) and established that breastfeeding, soy intake (including soy milk), and physical activity are key modifiable factors in the management of risk for breast cancer. The World Cancer Research Fund International supports the possible link between consuming soy foods and improved breast cancer prognosis [93].

In contrast to Western populations, Asian men have lower rates of prostate cancer development, similar to the pattern observed in breast cancer. High soy consumption has been proposed as a potential factor contributing to a significant reduction of up to 50% in prostate cancer risk. However, it is important to note that most of the available data on this association come from case–control studies rather than longitudinal studies [69]. Intervention studies showed that punctual exposure to isoflavones slows the rise in prostate-specific antigen (PSA) levels [94], whereas long-term trials were not efficient in avoiding the recurrence of prostate cancer after complete prostatectomy or the progression of advanced-grade neoplasia [95]. The studies examining the effects of isoflavones on prostate carcinoma have certain limitations in their design, such as variations in the administered dose of isoflavones or the concurrent use of other antioxidants. These limitations restrict the strength of the evidence supporting the findings. While the mechanism of action of these molecules in prostate carcinoma is not fully understood, some evidence suggests that they have the potential to inhibit metastasis. Moreover, genistein has been demonstrated to have chemopreventive effects through binding to the estrogen receptor β, which is expressed in prostate epithelial cells and has an important role in cellular homeostasis due to anti-proliferative [96], pro-differentiative [97], and pro-apoptotic actions [98]. In the particular case of soy milk, few investigations have been addressed to understand its role in prostate cancer incidence. Jacobsen et al. [66] carried out a prospective study with 225 cases of this pathology among 12,395 men who had declared their soy milk consumption through a food frequency questionnaire. They concluded that frequent consumption of soy milk (more than once a day) was associated with a 70% reduction in the risk of prostate cancer. Updated research needs to be conducted to increase the reliability of these findings.

While the scientific evidence is limited, there have been documented cases suggesting that soybeans and soy-derived foods may have an impact on other types of cancer as well. Apprey et al. [99] reported the anti-proliferative effects of isoflavonoids isolated from soy milk on lymphoma (DG 75) and leukemia (CEM) cell lines. Studies in animal models have proven that dietary protease inhibitors of the Bowman–Birk family (BBI) from several legume sources, including soybean, can prevent or suppress carcinogenic and inflammatory processes within the gastrointestinal tract. Although the therapeutic targets and the action mechanism of BBI have not yet been elucidated, the emerging evidence suggests that BBI proteins exert their preventive properties via protease inhibition [100]. The high amounts of hydrophilic phenolic compounds, saponins and phytates, present in soybean and other legumes might act as inhibitors for metalloproteinases, which are critical for the metastatic progression of colorectal cancer. Concerning endometrial tissue, for some years, the effect of soy products, including soy milk, on the promotion of endometrial hyperplasia has been questioned. This is a matter of concern taking into account the use of soy derivatives as hormone replacement therapy for the symptomatic treatment of menopause. Mahady [101] suggested that soy isoflavones did not cause endometrial hyperplasia when used in normal therapeutic doses. In fact, for premenopausal women, the data actually suggested that soy intake may protect against endometrial hyperplasia and cancer because soy isoflavones, in the presence of estrogens, appear to act as anti-estrogens. On the other hand, soy milk is theoretically an acceptable dietary option in patients with chronic liver failure due to the high content of vegetable protein and low content of methionine. The recent Japan Public Health Center-based prospective study recruited more than 70,000 participants to investigate the impact of soy intake on the risk of liver cancer, establishing no association between them [102].

It is important to note that the protective role of proteins and isoflavones in soy milk against cancer is still an area of ongoing research. The specific mechanisms and optimal dosages for cancer prevention are yet to be fully elucidated.

### 5.4. Menopause

In the early 2000s, a survey among peri-menopausal and menopausal women found that 79% of US women used botanical dietary supplements. Among those, 65% reported using these supplements to alleviate menopausal symptoms such as hot flashes, joint pain, insomnia, depression, anxiety, or fatigue. Interestingly, about 42% of the women in this survey included soy products, such as soy milk, soy proteins, and soy isoflavones, in their dietary regimen [103]. Their efficiency seems to be associated with the capability of soy isoflavones (including genistein, daidzein, and glycitein), which have a similar chemical structure to 17-β estradiol, to bind to estrogen receptors and mimic their effects [104]. A meta-analysis provided strong support for the efficacy of soybean isoflavones, which significantly reduced the frequency and severity of hot flashes by 20.6% and 26.2%, respectively [105]. A detailed revision of data revealed that in those trials providing genistein supplements (>18.8 mg/d), the reduction in hot flash frequency was more than twice that observed in those having lower genistein consumption. It was established that a dose of ≈40 mg of total isoflavones from soybeans was an efficient amount to control these manifestations. Tranche et al. [67] conducted a clinical assay in 147 peri- and postmenopausal women with the aim of evaluating the effects of a soy drink with a high concentration of isoflavones on climacteric symptoms. The experimental design was an open-label, controlled, crossover trial with intervention phases of 12 weeks (control or soy milk; 500 mL per day provided 15 g of protein and 50 mg of isoflavones) separated by a 6-week washout period. A reduction in climacteric and urogenital domain symptoms of 20.4% and 21.3%, respectively, was detected. Women also declared an improvement in their health-related quality of life by 18.1% through menopause rating scale and quality of life questionnaires. Another randomized trial established that the soy drink was only efficient for controlling the vasomotor alterations associated with menopause in women affected by the most severe symptoms, a situation in which daily consumption of 350 mL of a beverage containing 35 mg of isoflavones was enough to improve the signs [68].

### 5.5. Bone Health

Postmenopausal women experience a significant loss of bone mass due to decreased estrogen levels, resulting in an increased risk of fractures. Traditional treatments, such as estrogen therapy, have been shown to effectively manage bone loss and reduce hip fracture risk by one-third. In order to promote bone health in postmenopausal women, it is commonly recommended to consume soy foods or receive medical prescriptions for drugs containing synthetic isoflavones, as they have estrogen-like effects that can support bone health [69]. The mechanism behind this suggests a favorable action of isoflavones on bone turnover and on bone mineral density [106]. According to a prospective epidemiological study involving a significant number of vegetarian women in the US (40%), it was found that those who consumed soy milk at least once a day had 56% lower odds of developing osteoporosis compared to non-consumers. The authors concluded that soy milk consumption exhibited a protective effect against osteoporosis development [107].

Apart from menopause, fortified soy milk consumption has been described to have a positive influence on other life stages. In a study conducted by Ho et al. [108] the impact of one-year supplementation with calcium-fortified soy milk on the bone health of Chinese adolescent girls aged 14-16 was examined. The findings suggested that consuming 375 mL of the calcium-fortified beverage was significantly correlated with a higher percentage of bone mass density and content at the hip. These results indicate that this approach could be an effective strategy for promoting bone acquisition and optimizing peak bone mass in adolescent girls. In the same line, a short trial developed in 10 healthy subjects (22.4 ± 1.2 years old) reported that calcium-fortified soy milk might be effective for maintaining vascular and bone health [109].

In summary, the effects on bone health are complex. Factors such as calcium content, mineral bioavailability, and overall dietary considerations play a role in assessing its effects. It is noteworthy that many studies demonstrating positive effects on bone health involve the consumption of calcium-fortified soy milk.

### 5.6. Mental Health and Cognitive Impairment

Several studies have attempted to explore the potential mental health effects of soy milk, suggesting that it may have similar but less pronounced impacts compared to soy isoflavones. However, definitive evidence establishing these effects has not yet been established [68]. Some of the mental illnesses where soy milk consumption could have some impact include depression in postmenopausal women, due to the presence of isoflavones able to counteract the deficit of reproductive hormones, a fact which seems to be involved in the etiology of this disorder [69]. Due to its isoflavone content, soy milk consumption has been suggested to potentially reduce the risk of cognitive decline and dementia, since these molecules have demonstrated their suitability in several Alzheimer’s disease-like pathologies, including removing amyloid β protein and decreasing tau phosphorylation [110]. Some studies investigating the impact of soy milk and its isoflavones on the psychological well-being and mood stability of peri- and postmenopausal women have reported no significant effects following a dietary intervention with soy milks containing varying amounts of soy isoflavones (10, 35, or 60 mg/day) [104]. More research is necessary to further explore the relationship between soy milk consumption and mental health.

### 5.7. Gut Health

The mucosal surface of the intestinal tract is a complex ecosystem formed by the gastrointestinal epithelium, immune cells, and resident microbiota. In response to various injuries, the body generates a protective immune response through the production of mediators such as cytokines, growth factors, and adhesion molecules. These mediators play a crucial role in initiating and amplifying the inflammatory response, which is necessary for safeguarding the intestine against attacks from pathogens. However, an excessive production of inflammatory mediators can disrupt the balance of the bowel epithelium and lead to intestinal imbalances. Inflammatory bowel disease (IBD), including Crohn’s disease and ulcerative colitis, are chronic inflammatory conditions of the gut with complex causes that currently have no cure. However, the use of agents that inhibit the inflammatory response can significantly improve the symptoms and outcomes of these diseases [111]. Isoflavones, especially genistein, daidzein, and its active metabolite equol, have been proposed as active molecules for the treatment of IBD due to their anti-inflammatory ability, although the molecular mechanisms explaining the suppression of the inflammatory response are not yet well-elucidated. Calvello et al. [111] analyzed the effects of commercial bovine and soybean milks and their bioactive compounds, namely genistein, daidzein, and equol, on the inflammatory responses induced by lipopolysaccharide treatment of human intestinal Caco-2 cells in terms of nitric oxide (NO) release and inducible nitric oxide synthetase (iNOS) expression. These authors demonstrated that soy beverages and isolated compounds were able to reduce inflammatory responses from intestinal cells thanks to the downregulation of iNOS expression, decreasing NO production. The action seemed to be directly attributable to the selective inhibition of the NF-kB signaling pathway, providing the first molecular description of the anti-inflammatory properties of isoflavones. A randomized clinical trial involving thirty patients with ulcerative colitis was conducted to assess the effects of soy milk supplementation (250 mL/day) alongside routine treatments compared to routine treatments alone over a period of 4 weeks. The results indicated that the anti-inflammatory action observed was not solely attributed to the presence of isoflavones but also to specific proteins present in soy milk [112]. Data suggest that soy milk consumption is capable of decreasing intestinal inflammation, inducing changes in gut microbiota composition, increasing the abundance of beneficial/commensal bacteria, and reducing the mucus-degrading taxa.

Beneficial effects of soy milk consumption on gut health have also manifested in other pathologies. Fernandez-Raudales et al. [113] examined the effect on gut microbiota in overweight and obese men after drinking a daily soy milk supplement (500 mL). There was an increase in total bacteria, and a notable modulatory effect on the gut microbiota was observed, mediated by a decrease in the abundance of bifidobacteria and Firmicutes, while Bacteroidetes and Proteobacteria increased. The shift in the ratio of Firmicutes to Bacteroidetes has been linked to positive health outcomes, such as a reduced risk of obesity and other metabolic syndromes.

The capability of soy milk to modify gut microbiota in healthy subjects has been also documented. Fujisawa et al. [114] established that the number of fecal bifidobacteria significantly increased after the intake of 100 mL of soy milk once a day in addition to the normal diet for two consecutive weeks; this seems to be related to the reported prebiotic properties of soybean oligosaccharides.

### 5.8. Other Actions

Several investigations suggest that isoflavones help to reduce wrinkles and other external aging manifestations [69], but little information is available for soy beverages. In a 14-week trial involving 159 postmenopausal women, the consumption of a beverage containing isoflavones, along with other bioactive compounds, resulted in a significant improvement in the depth of facial wrinkles. This improvement was clinically measurable and was associated with an increased deposition of new collagen fibers in the dermis [115].

Due to shifts in hormonal status and aging, postmenopausal women become susceptible to chronic diseases mediated by immune dysfunction and oxidative stress. Ryan-Borchers et al. [116] demonstrated that intake of 700 mL of soy milk/day was able to stimulate B cells and inhibited DNA oxidative damage in postmenopausal women aged 50–65 years due to the presence of isoflavones. However, the consumption of the beverage did not have a significant impact on the concentrations of interferon γ, interleukin 2, TNFα, C-reactive protein in plasma, or 8-isoprostane in urine.

### 5.9. Controversial Effects

In comparison to cow milk, most vegetable beverages generally contain lower levels of fat and proteins, which also have a lower biological value than casein. This disparity in nutritional composition may account for the observed negative association between soy beverage consumption, either in isolation or combined with traditional milk, and height. A cross-sectional study called TARGetKids!, which involved 5034 healthy Canadian children aged 24 to 72 months, found a dose-dependent relationship between increased consumption of non-cow’s milk and shorter height. For each daily cup of non-cow’s milk consumed, children were on average 0.4 cm shorter [117]. However, full-term neonates fed with soy-based infant formula during the first three months of life showed normal growth during the initial three years, similar to infants fed with breast milk or casein-based formula [118]. This suggests that the more balanced nutritional profile of soy-based infant formula compared to soy milk may have contributed to their normal growth pattern during this crucial period. Recently, the normal pattern of growth of Indonesian infants with a cow milk allergy and fed with soy-based infant formula has been demonstrated [119].

The behavior of milk isoflavones as phytoestrogens has raised the possibility of an effect on male fertility, which is coincident with the growing apprehension that environ-mental estrogens may be linked to the declining sperm count observed in men worldwide [120]. Two case–control studies have reported isolated cases where individuals exhibited feminizing effects, such as erectile dysfunction, increased estrogen levels, loss of libido, gynecomastia, and low testosterone, in response to excessive intake of isoflavones (360 mg/day) [121,122]. Chavarro et al. [123] conducted a small pilot cross-sectional study that found a modest association between soy food consumption, including soy milk, and lower sperm concentration. However, subsequent studies by the same research group reported no significant relationship between the consumption of soy foods and various reproductive outcomes, including fertilization rates, proportions of poor-quality embryos, rates of embryo cleavage, implantation, clinical pregnancy, and live births [124]. In the same line, a recent meta-analysis of 41 clinical trials, predominantly conducted in Western populations, found no significant effects of soy or isoflavones on reproductive hormone levels in men. This analysis suggests that soy consumption does not have a substantial impact on male reproductive hormones based on the collective evidence from various studies [125].

The potential effects of excessive intake of isoflavones on male feminization have raised concerns in recent years. A recent case report study documented a case of secondary hypogonadism resulting from the daily consumption of 1.2 L of soy milk (equivalent to approximately 310 mg of isoflavones) over a period of 3 years. This excessive intake led to low levels of gonadotropins and testosterone, accompanied by symptoms such as erectile dysfunction and gynecomastia [126]. Despite concerns about the potential development of gynecomastia (enlargement of male breast tissue) due to soy consumption, two placebo-controlled clinical trials involving 200 men administered 66 mg/d of isoflavones (equivalent to approximately 250 mL of soy milk) for 3 months and more than 300 men who received close to 100 mg/d for 3 years, respectively, did not exhibit gynecomastia [127,128]. 

Two decades ago, in vitro studies demonstrated that isoflavones had the potential to serve as an alternative substrate to tyrosine for iodination. This finding raised concerns that when iodine intake is insufficient, isoflavones could potentially worsen thyroid function. Furthermore, these studies also revealed that isoflavones inhibited the activity of thyroid peroxidase (TPO), both in vitro and in rats. These findings suggested that isoflavones might have an impact on thyroid function and metabolism, particularly in situations where iodine availability is inadequate [129]. TPO is an enzyme necessary for the production of thyroxine (T4) and triiodothyronine (T3), which are essential thyroid hormones [130]. However, the meta-analysis of 18 clinical trials carried out by Otun et al. [131] investigating the effect of soy and isoflavones on thyroid hormones found no effect on free levels of T4 or T3 and a very modest increase in TSH level (Thyroid-Stimulating Hormone) with unclear clinical relevance. On its part, EFSA has concluded that isoflavone supplements do not affect thyroid function in postmenopausal women [132].

Given the structural similarity between isoflavones and phytoestrogens, it was postulated that isoflavones could potentially influence female estrogen levels [133,134], but there is currently no clear evidence supporting this claim. In fact, the meta-analysis by Hooper et al. [135] based on a clinical trial in post- and premenopausal women concluded that there were no effects of soy or isoflavone intake on estradiol, estrone, sex hormone binding globulin, follicle-stimulating hormone (FSH), or luteinizing hormone (LH).

Concerning the effects of soy food consumption and puberty onset, evidence clarifying this possible association is limited. Indeed, an early onset of puberty is occurring, but this is happening both in countries with high intake of soy derivatives and in those with little tradition of consumption [128]. Clinical trials conducted on girls aged 5–14 years in the United States, where they were administered 18–46 mg of isoflavones for 8 weeks, revealed no significant effects on hormone levels [136,137]. Additional observational studies and clinical research are needed in this matter.

## 6. Conclusions

This review provides an overview of a market survey conducted on soy drinks available in Spain and other EU countries. Fortified soy drinks have emerged as the dominant choice in the Spanish and EU market. By fortifying these beverages, the nutritional gap with cow’s milk is successfully bridged, making fortified soy drinks the preferred choice among consumers seeking a wholesome alternative to traditional dairy products. We have compiled a broad list of advancements in processing technologies that are specifically designed to enhance the final quality of soy milk products. Additionally, we have gathered valuable information regarding the nutritional composition. Numerous studies and meta-analyses have highlighted the positive effects of soy drinks on various health conditions, including cardiovascular-related diseases, hormone-dependent cancers, menopause, and bone, mental, and gut health. The primary drivers of these beneficial actions have been identified as isoflavones and their metabolites, but the contribution of specific proteins is also important. In the upcoming years, ongoing research will contribute significantly to our understanding of the impact of these beverages on various aspects such as growth during early life stages, puberty onset, fertility, feminization, and the thyroid gland. This research will address the current controversies surrounding health claims associated with soy milk, providing valuable insights and a clearer understanding of its effects. By shedding more light on these areas of investigation, future research will help establish a more comprehensive and evidence-based understanding of the potential health implications of soy milk consumption.

## Figures and Tables

**Table 1:** Nutritional composition of commercial soy beverages found in the Spanish market as described in the labelling and sorted by category.

Category	Original (*n* = 7)		Fortified (*n* = 31)		Sugar-Free (*n* = 20)		Light (*n* = 4)		Flavored (*n* = 13)	
	Mean Values (*n*)	Range	Mean Values (*n*)	Range	Mean Values (*n*)	Range	Mean Values (*n*)	Range	Mean Values (*n*)	Range
% Soybeans	11.4	7.4–16.0	10.9	5.0–14.5	11.5	5.5–15.0	7.6	5.0–11.0	10.3	7.2–13.0
Nutritional values	*per 100 mL*									
Energy (kJ)	174	148–206	187	96–299	149	96–205	117	113–124	262	160–320
(kCal)	42	36–49	44.6	23–71	35.6	23–49	28	27–30	62	38–76
Fats (g)	1.9	1.7–2.3	1.8	1.1–2.9	1.9	1.20–2.9	1.2	1.1–1.4	2.1	1.6–2.9
Saturates	0.4	0.3–0.6	0.3	0.2–0.6	0.3	0.2–0.6	0.2	0.2–0.3	0.4	0.3–0.7
Mono-unsaturated	0.6 (3)	0.4–1.1	0.5 (18)	0.2–1.1	0.4 (7)	0.4–0.5	0.3 (3)	0.2–0.3	0.5 (8)	0.3–0.7
Polyunsaturated	1.1 (3)	1.0–1.1	1.1 (18)	0.7–1.7	1.1 (7)	1.0–1.3	0.7 (3)	0.7–0.7	1.2 (8)	0.9–1.7
Carbohydrate (g)	2.5	0.8–4.0	3.6	0–9.2	1.3	0–7.0	1.6	0.9–2.0	6.8	2.5–10.1
Sugars	1.9	0.5–3.2	2.9	0–7.6	0.7	0–3.2	1.5	0.6–2.0	6.3	1.7–9.5
Fiber (g)	0.6 (4)	0.4–0.8	0.6 (24)	0.3–1.3	0.6 (9)	0.3–0.9	0.8 (3)	0.5–1.2	0.9 (10)	0.4–1.3
Protein (g)	3.4	3.0–3.7	3.2	2.1–6.0	3.2	2.1–3.8	2.3	2.1–2.6	3.6	3.0–6.0
Salt (g)	0.11	0.05–0.20	0.10	0–0.20	0.08	0.02–0.18	0.11	0.09–0.14	0.12	0.04–0.2
Minerals	*per 100 mL*									
Calcium (mg)	133 (4)	120–150	123 (31)	60–150	108 (6)	60–120	135 (4)	120–140	126 (7)	120–150
Potassium (mg)	-	-	52 (1)	-	-	-	-	-	-	-
Phosphorus (mg)	-	-	70 (2)	70–70	70 (1)	-	-	-	-	-
Magnesium (mg)	-	-	70 (1)	-	70 (1)	-	-	-	-	-
Iron (mg)	-	-	2.50 (1)	-	-	-	-	-	2.5 (1)	-
Vitamins	*per 100 mL*									
Vitamin A (µg)	120 (2)	120–120	118 (16)	80–120	100 (2)	80–120	120 (1)	-	120 (3)	120–120
Vitamin D (µg)	0.79 (4)	0.75–0.90	0.82 (25)	0.75–2.00	1.17 (3)	0.75–2.00	0.75 (2)	0.75–0.75	0.79 (6)	0.75–1.00
Vitamin E (µg)	-	-	1.60 (1)	-	1.60 (1)	-	-	-	-	-
Vitamin C (mg)	-	-	10 (1)	-	10 (1)	-	-	-	-	-
Thiamine (mg)	-	-	0.20 (1)	-	0.20 (1)	-	-	-	-	-
Riboflavin (mg)	0.21 (1)	-	0.21 (8)	0.21–0.21	0.21 (1)	-	0.21 (1)	-	0.21 (3)	0.21–0.21
Niacin (mg)	-	-	1.60 (1)	-	1.60 (1)	-	-	-	-	-
Vitamin B6 (mg)	-	-	0.12 (1)	-	0.12 (1)	-	-	-	-	-
Folic acid (µg)	-	-	65 (1)	-	65 (1)	-	-	-	-	-
Vitamin B12 (µg)	0.38 (1)	-	0.38 (9)	0.38–0.40	0.38 (1)	-	0.38 (1)	-	0.39 (4)	0.38–0.40

Figures in brackets indicate the number of drinks that declared that nutrient in the labelling. When not specified, the ingredient was present in all drinks.

**Table 2:** Novel processing techniques addressed to improve the nutritional quality of soy beverages.

Processing Technique	Effect on Nutritional Quality	References
Manothermosonication	Inactivation of LOX, POD, and TI	[21]
Ultrasound	Increase in product stability Increase in protein, oil, and solid extraction yield Reduction in off flavor Reduction in microorganisms’ total number Applied as a pre-treatment to soybeans: increase in isoflavone content	[20,22,23,24]
Microwave	Decrease in LOX and TI activities	[17]
Microwave and ultrasound	Increase in protein and fat content	[25,26]
Low-intensity Pulsed Electric Fields	Modification of isoflavone profile and content	[27]
Enzymatic pre-treatments	Reduction in okara residues Increase in protein and isoflavone content	[28]
Radio-frequency pre-treatment	Improvement of sensorial properties Decrease in LOX, urease, and TI activities	[29]
Dielectric barrier discharge plasma	Reduction in TI activity	[30]
High-temperature pressure cooking	Improvement of digestibility Improvement of sensorial properties	[31]
Addition of natural compounds: tea polyphenols, steviose	Inactivation of TI	[32,33,34]
Immobilized Phytase System	Phytic acid reduction	[35]

**Table 3:** Human studies supporting beneficial effects of soy milk consumption.

Pathology	Study Design	Target Population	Observed Effect	References
Cardiovascular risk	Interventional study supplying 250 mL/d soy milk for 28 days	Healthy Nigerian (18–35 y) (*n* = 39; 18 men, 21 women)	Increase in serum antioxidant capacity and glutathione S-transferase activity. Decrease in malondialdehyde	[62]
	Interventional study supplying 1L/d soy milk or cow milk for 3 months	Spanish moderate hypertensive patient (18–70 y) (*n* = 40; 25 men, 15 women)	Hypotensive action which correlated with genistein urinary excretion	[63]
Breast cancer	The Adventist Health Study: prospective study with dietary questionnaires over 7.9 years	American women free of cancer (>30 y) (*n* = 52,795)	Substitution of dairy product with soy milk decreased the risk of breast cancer development	[64]
	Unmatched hospital-based case–control study: patient monitored with dietary questionnaires	Malaysian women (*n* = 4663; 3683 cases, 3980 controls)	Breastfeeding, soy intake (including soy milk), and physical activity helped in managing breast cancer risk	[65]
Prostate cancer	The Adventist Health Study: Prospective study among soy milk consumers using food frequency questionnaires	American men (≥25 y) (*n* = 12,395; 225 cases)	Soy milk consumption higher than once a day was associated with 70% reduction in prostate cancer risk	[66]
Menopause	Open-label, controlled crossover trail with intervention phases of 12 weeks (500 mL control/soy milk with 15 g protein and 50 mg isoflavones)	Spanish peri- and post-menopausal women (>45 y) (*n* = 147)	Reduction in climacteric (20%) and urogenital (21%) symptoms. Improvement in health-related quality of life	[67]
	Randomized 12-week interventional trial supplying soy milk high, medium, or low in isoflavones	UK post-menopausal women (44–63 y) (*n* = 101)	350 mL/d soy milk containing 35 mg isoflavones decreased vasomotor symptoms	[68]

## Data Availability

Data related to the market survey included in this study are available on request. The rest of the data were not created or analyzed in this study.
